# 3D alveolar organoid drug screening model for targeting TGF-β1 in pulmonary fibrosis

**DOI:** 10.1016/j.bbrep.2025.102191

**Published:** 2025-08-11

**Authors:** Hyeong-jun Han, Hyunyoung Kim

**Affiliations:** a Division of Intractable Diseases, Department of Chronic Diseases Convergence Research, Korea National Institute of Health, Cheongju, 28160, Republic of Korea; b National Stem Cell Bank of Korea, Korea National Institute of Health, Cheongju, 28160, Republic of Korea

**Keywords:** Alveolar type II organoid, TGF-β1, Pulmonary fibrosis

## Abstract

Idiopathic pulmonary fibrosis (IPF) is a prototype of chronic, progressive, and fibrotic lung disease. Excessive deposition of extracellular matrix (ECM) results in fibrotic remodeling, alveolar destruction, and irreversible lung dysfunction. In addition to myofibroblast activation and ECM deposition, repetitive lung epithelial cell damage and reprogramming areconsidered to be closely involved in IPF pathogenesis. Transforming growth factor (TGF)-β1 plays an important role in IPF and cancer; it is a major pro-fibrotic cytokine, and is a potential target for treating fibrotic diseases.TGF-β1 binds to TGF-βRII, phosphorylating TGF-βRI, and enhances ECM expression via the suppressor of mothers against decapentaplegic (SMAD) phosphorylation signaling pathway. Current medical interventions for IPF are predominantly anti-fibrotic medications such as pirfenidone and nintedanib, which are effective in delaying lung function deterioration, reducing acute symptom exacerbations, and increasing overall life expectancy. However, these pharmaceutical agents cannot repair fibrotic pulmonary tissues or impede disease progression. To bridge this gap, we constructed a model of TGF-β1-induced fibrosis and screened for potential drugs. From 320 anti-fibrotic drugs, 9 hits were found in the TGF-β1-induced fibrosis model, and after validation, the final 7 hits were identified as TGF-β1 inhibitors. All the 7 hits were confirmed as TGF-βRI inhibitors, which showed that the model could quickly and easily discover new compounds that can act as TGF-β1 inhibitors.

This study is significantbecausewe useda 3D model to swiftly and precisely identify TGF-β1 inhibitors, potentially accelerating the clinical translation of TGF-β1-targeted therapies for fibrotic diseases.

## Highlights

• Transforming growth factor (TGF)-β1 is a potential target for treating fibrotic diseases.
• 3D alveolar organoid drug screening model was used to swiftly and precisely identify TGF-β1 inhibitors.
• This model accelerates the clinical translation of TGF-β1-targeted therapies for fibrotic diseases.

## 1 Introduction

Lung organoid systems have become valuable tools for investigating developmental organogenesis, diseases, and regeneration [1]. Human primary pulmonary alveolar epithelial cells include alveolar epithelial type II (AEC2) and alveolar epithelial type I (AEC1) cells [2]. AEC1 cells are the major gas exchange cells, whereas AEC2 cells are the progenitors of AEC1 cells and are responsible for surfactant synthesis and homeostasis after injury [3]. AEC2 cells differentiate into AEC1 [4] and can self-renew and replace damaged AEC1 cells to restore alveolar integrity after an injury [5].

Idiopathic pulmonary fibrosis (IPF) is a proto type of persistent, progressive, fibrotic lung disease [6]. Identifying the underlying mechanisms of IPF is essential for understanding the crucial events that trigger unusual fibroblast outgrowth, progressive tissue scarring, and/or abnormal epithelial repair [7]. Healthy tissue is exchanged by an altered extracellular matrix (ECM), and the alveolar architecture is devastated, which leads to reduced lung compliance, disrupted gas exchange, and ultimately respiratory failure and death. Excessive ECM deposition results in fibrotic remodeling, alveolar destruction, and irreversible lung dysfunction. In addition to myofibroblast activation and ECM deposition, repetitive lung epithelial cell damage and reprogramming are considered to be closely involved in IPF pathogenesis [6,8]. Currently, two drugs, nintedanib and pirfenidone, are approved for treatment; however, they only reduce the rate of lung function decline and new therapeutic options are needed [9]. Transforming growth factor (TGF)- β is a major pro-fibrotic cytokine, which promotes the formation of myofibroblasts involved in the induction of lung fibrosis [10]. Therefore, it is a promising potential target for treating fibrotic diseases [11].

TGF-β signaling plays a critical role in a variety of biological processes and performs various functions such as cell growth inhibition, apoptosis, differentiation, and epithelial-mesenchymal transition (EMT) [12]. The TGF-β signaling cascade is tightly regulated and plays a critical role in the maintenance of cellular homeostasis,and development and organogenesis. Therefore, disruption of TGF-β signaling leads to life-threatening diseases such as cancer, fibrosis, and congenital malformations.TGF-β1 is a subtype of the TGF-β family (TGF-β1, TGF-β2, and TGF-β3) that plays a crucial role in the development of fibrosis and solid tumor.Structures of TGF-β1, TGF-β2, and TGF-β3 are highly similar and have closely related ligands [13,14]. TGF-β1 binds to type II receptor (TGF-βRII) and heterodimerizes with type I receptor (TGF-βRI) [15]. TGF-β1 exerts its biological effect through binding of the TGF-βRI and TGF-βRII. The binding of TGF-β1 to TGF-βRII induces the phosphorylation of serine and threonine residues located in TGF-βRI, resulting in a conformational change of TGF-βRI and subsequent activation of suppressor of mothers against decapentaplegic (SMAD) signaling pathways [16]. Heteromeric complexes with SMAD2, SMAD3, and SMAD4 are formed by phosphorylated SMAD proteins [17]. This complex translocates into the nucleus to activate EMT by inducing the expression of several mesenchymal transcription factors, such as Snail and Twist, and affects the transcription of genes involved in the fibrotic response [18].

In general, pharmacological approaches to treat IPF have focused on altering fibroblast pathways. However, IPF, by far the most aggressive fibrotic lung disorder, differs from the other ones due to the leading role of lung epithelium in its pathogenesis. TGF-β1 signaling has been particularly identified as a promising target for fibrotic diseases [11].

The significance of this research lies in its innovative approach to modeling IPF using human lung organoids, which more accurately represent the human lung microenvironment compared to traditional two-dimensional (2D) cell cultures. This advancement allows for more precise evaluation of drug efficacy and safety in a setting that closely mimics *in vivo* conditions. Furthermore, the identification of TGF-β1 inhibitors through this platform highlights the potential for discovering novel therapeutic agents that can specifically target epithelial cell dysfunction in IPF, addressing a critical gap in current treatment strategies. By focusing on the epithelial component of IPF pathogenesis, this study offers a complementary approach to existing therapies that primarily target fibroblast activity, thereby broadening the scope of potential interventions for this debilitating disease.

## 2 MATERIALS and METHODS

### 2.1 Reagents

mTeSR™1 Complete Kit, Stemdiff ™ Definitive Endoderm Differentiation Kit, and cloneR were supplied by Stem Cell Technologies (Vancouver, Canada; 85850 and 05110). Iscove's Dulbecco's modified Eagle medium (DMEM) and Ham's F-12 medium containing l-glutamine were supplied by Corning Incorporated (Corning, NY,USA; 10-016-CV, 10-080-CV). N2, B27, bovine albumin fraction V, penicillin–streptomycin, and GlutaMAX were supplied by Gibco (Grand Island, NY, USA; 17502-048, 17504-044, 15260037, 15140122, and 35050061). l-ascorbic acid, 1-thioglycerol, dorsomorphin, dexamethasone, IBMX, and 10 μM SB431542 were supplied by Sigma-Aldrich (St.Louis, MO, USA; A4544, M6145, P5499, D4902, I5879, and S4317). BMP4 was supplied by R&D Systems (Minneapolis, MN, USA; 345-FG). CHIR99021, 8-bromo-cAMP, and retinoic acid were supplied by Tocris (Bristol, UK; 4423, 1140, and 0695). FGF7 was supplied by Peprotech (Rocky Hill, NJ, USA; 100-19). Accutase was purchased from Merck (Rahway, NJ, USA; SCR005).The antifibrosis compound library was supplied by TargetMol Chemicals, Inc. (Boston, MA, USA).

### 2.2 Cell culture

The WA09 (H9) human embryonic stem cell line was obtained from WiCell (Madison, WI, USA) and cultured on Matrigel-coated dishes inmTeSR™1. ifibroblasts were cultured in a FibroLife S2 Medium (Lifeline Cell Technology, Walkersville, MD).

### 2.3 Generation of alveolar type II organoids

To differentiate WA09 (H9) cells into hiAT2 organoids, we modified a previously reported protocol [5]. The steps involved are as follows.

**Day 0–Day 2**: WA09 (H9) cells were dissociated into single cells with Accutase and plated on Matrigel-coated dishes in mTeSR™1 adding cloneR. The cells were differentiated into definitive endoderm (DE) using a Stemdiff™ Definitive Endoderm Differentiation Kit.

**Day 3–Day 5**: DE cells were further differentiated into anterior foregut endoderm by adding serum-free differentiation (SFM) medium composed of 75 % Iscove's DMEM, 15 % Ham's F-12 medium with l-glutamine, 0.5 × N2, 0.5 × B27, 7.5 % bovine albumin fraction V, 1 % penicillin–streptomycin, 1 % GlutaMAX, 50 μg/ml l-ascorbic acid, and 0.4 μM 1-thioglycerol, supplemented with 2 μM dorsomorphin and 10 μM SB431542.

**Day 6–Day 14**: The differentiated cells were further differentiated into lung progenitor cells by treating with SFM medium containing 10 ng/ml BMP4, 3 μM CHIR99021, and 100 nM retinoic acid.

**Day 15–Day 30**: CD47^high^ CD26^low^ cells were sorted and replatedin Matrigel (200 cells/μl) to enrich in NKX2-1+ lung progenitors. The cells were differentiated into alveolospheres in SFM medium supplemented with 3 μM CHIR99021, 10 ng/ml FGF7, 50 nM dexamethasone, 100 μM 8-bromo-cAMP, and 100 μM 3-isobutyl-1-methylxanthine (IBMX). To improve the efficiency of surfactant protein C (SFTPC), CHIR99021 was withdrawn for 5 days and added back. The alveolospheres were passaged every 14 days.

### 2.4 Cell viability assay using Cell counting Kit-8 (CCK-8)

Organoid viability was measured using the CCK-8 assay (Dojindo, Tokyo, Japan). Briefly, the organoids were seeded in 96-well plates and cultured for 2 days. Organoids were treated with 50 ng/ml TGF-β1, with or without antifibrotic drugs treatment for 3 days. Then, 10 μl CCK-8 solution was added per well and incubated for 3 h. The absorbance at 450 nm was measured using a microplate reader (Biotek, Winooski, VT, USA).

### 2.5 Gel contraction assay

To differentiate iFibroblasts, we followed a previously reported protocol [19]. iFibroblasts were counted using a Cell Counter. iFibroblasts were prepared at 2x10^5^ cells/ml in 20 μl Geltrex droplets in a 35-mm dish. The solution was polymerized at 37 °C in 5 % CO_2_ incubator for 30 min. After polymerization, the gels were gently released from the dish, which contained 2 mL of fibroblast medium with or without TGF-β1 and anti-fibrotic drugs. The area of each gel was measured using an image analyzer.

### 2.6 Western blot assay

The cells were lysed in radioimmunoprecipitation assay (RIPA) lysis buffer (Thermo Fisher Scientific, Waltham, MA, USA) containing a protease inhibitor (Roche, Basel, Switzerland). The protein concentration was measured using a bicinchoninic acid (BCA) Protein Assay kit (Thermo Fisher Scientific, Waltham, MA, USA), and similar amounts of protein were separated by sodium dodecyl sulfate-polyacrylamide gel electrophoresis (SDS-PAGE). The proteins were transferred to polyvinylidene difluoride (PVDF) membranes (Thermo Fisher Scientific, Waltham, MA, USA) and blocked with Western Blocker™ Solution (Sigma, St. Louis, MA, USA) for 1 h at room temperature. After blocking, the membranes were incubated with primary antibodies, FN1, SMAD2, phospho-SMAD2, E-cadherin, N-cadherin, Vimentin, GAPDH (Cell Signaling Technology, Beverly, MA, USA), and β-actin (Sigma, St. Louis, MA, USA) overnight at 4 °C. The membrane was washed twice with 0.1 % tris-buffered saline with Tween® 20 (TBS-T) for 15 min and incubated with the secondary antibody for 1 h at room temperature. After washing twice with 0.1 % TBS-T for 15 min, the protein bands were detected using an Amersham Imager 600 (GE Life Sciences, Marlborough, MA, USA). The specificity of each primary antibody was validated by confirming that the detected band corresponded to the predicted molecular weight of the target protein, as stated in the antibody manufacturer's datasheet.

### 2.7 Immunostaining

Cells were fixed with 4 % paraformaldehyde (Wako, Osaka, Japan) for 20 min and permeabilized with 0.25 % Triton X. Fixed cells were incubated with primary antibodies against stage-specific embryonic antigen-4 (SSEA-4) (Millipore, Billerica, MA, USA), TRA-1-60 (Millipore, Billerica, MA, USA), TRA-1-81 (Millipore, Billerica, MA, USA), and octamer-binding transcription factor 4 (Oct4) (Santa Cruz, CA, USA). After 24 h, the samples were washed with 1x phosphate buffered saline (PBS) and incubated with fluorescein-conjugated secondary antibodies (Vector Laboratories, Burlingame, CA, USA). The nuclei were stained with 4′,6-diamidino-2-phenylindole (DAPI) (Thermo Fisher Scientific, Waltham, MA, USA). Finally, images were acquired using Olympus JP/IX83 and CellSens Software [20].

### 2.8 Real-time quantitative PCR (qRT-PCR) analysis

Total RNA was isolated using the RNeasy Mini Kit (QIAGEN, Hilden, Germany) according to the manufacturer's instructions. 1 μg RNA was used to cDNA synthesis. cDNA was synthesized using RNA to cDNA EcoDry Premix (Clontech, Mountain View, CA, USA). 1 μL cDNA solution among 10 μL was used to qRT-PCR analysis. qRT-PCR analyses were performed using the Taqman™ Master Mix (Applied Biosystems, Carlsbad, CA, USA) in a QuantStudio 6 Flex Real-Time PCR System (Applied Biosystems, Carlsbad, CA, USA). PCR products were validated by melt curve analysis to confirm amplification specificity. The melt curve was generated by slowly increasing the temperature from [60 °C] to [95 °C]. All quantitative gene expression data were normalized using a single, pre-selected housekeeping gene (*GAPDH*, *ActinB* or *HPRT1*) per target gene, not the geometric mean of these reference genes. Primer sequences are listed in Table 1. PCR products were validated by melt curve analysis to confirm amplification specificity.

**Table 1:** Nucleotide sequence of Primers used in qRT-PCR.

Gene	Sequence (5′-3′)	Tm (°C, Primer)	Tm (°C, Product)
*SFTPC*	5′-TTGGTCCTTCACCTCTGTCC-3′	58	53
	5′-CTCCCACAATCACCACGAC-3′	58	
*ABCA3*	5′-TTCTTCACCTACATCCCCTAC-3′	55	50
	5′-CCTTTCGCCTCAAATTTCCC-3′	56	
*FN1*	5′-CCACCCCCATAAGGCATAGG-3′	60	55
	5′-GTAGGGGTCAAAGCACGAGTCATC-3′	61	
*E-CAD*	5′-CCGCCTCCTTCTTCTCATCATAG-3′	59	54
	5′-GCTGCTCTTGCTGTTTCTTCG-3′	59	
*N-CAD*	5′-GCCCCTCAAGTGTTACCTCAA-3′	59	53
	5′-AGCCGAGTGATGGTCCAATTT-3′	58	
*VIM*	5′-AAGTTTGCTGACCTCTCTGAGGCT-3′	62	57
	5′-CTTCCATTTCACGCATCTGGCGTT-3′	62	
*ACTB*	5′-GAGCACAGAGCCTCGCCTTT-3′	62	57
	5′-ACATGCCGGAGCCGTTGTC-3′	63	
*GAPDH*	5′-CCCCACACACATGCACTTACC-3′	61	54
	5′-TTGCCAAGTTGCCTGTCCTT-3′	59	
*HPRT1*	5′-GACCAGTCAACAGGGGACAT-3′	59	50
	5′-CCTGACCAAGGAAAGCAAAG-3′	55	

### 2.9 Statistical analysis

Significant difference among the groups was determined at values of p < 0.05.

## 3 Results

### 3.1 Characterization of alveolar organoids

**Fig. 1:**
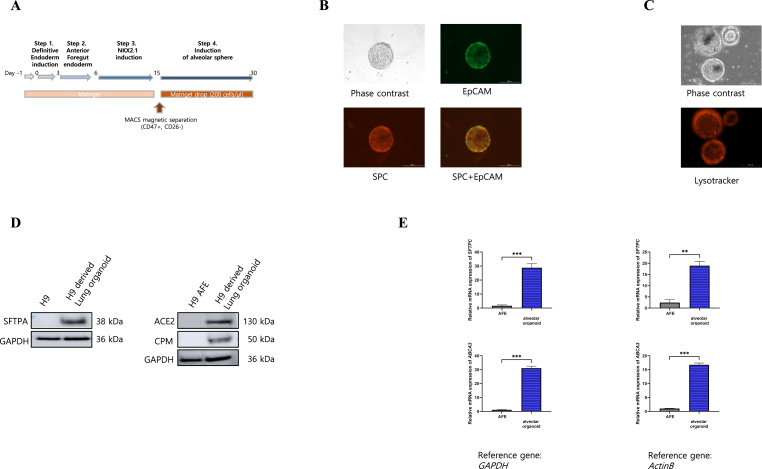
Characterization of alveolar organoids. (A) Schematic diagram illustrating the differentiation protocol. (B) Bright-field and immunofluorescent images of alveolar type II cell marker (SFTPC) and epithelial cell marker (EpCAM) in alveolar organoids. (C) Immunofluorescent image of alveolar type II cell marker, lysotracker. (D) Protein levels of SFTPA, ACE2 and CPM in alveolar organoids. (E) mRNA levels of *SFTPC* and *ABCA3.*

To produce alveolar organoids, we used the embryonic stem cell line WA09 (WiCell) and applied the differentiation protocol previously described by Jacob et al. [5]. The cells were adapted to Matrigel and differentiated into a definitive endoderm using the STEMdiff definitive endoderm kit. Definitive endoderm cells were replated and transferred to anterior foregut endoderm induction medium and lung progenitor induction medium, and the alveolar organoids were generated from a CD47^high^/CD26^low^ cell population. The cells were embedded in Matrigel to form alveolar type II-like cells (Fig. 1A). We confirmed the expression of surfactant protein C (SFTPC) (Fig. 1B) and lysotracker (Fig. 1C). In addition, we examined the expressions of surfactant apoprotein A (SFTPA), Carboxypeptidase M (CPM), and angiotensin-converting enzyme 2 (ACE2), which is severe acute respiratory syndrome coronavirus 2 (SARS-CoV-2) entry-related proteins (Fig. 1D). And we examined the mRNA expressions of *SFTPC* and *ATP-binding cassette A3* (*ABCA3*), which is a phospholipid transporter critical for surfactant homeostasis in pulmonary alverolar type II (Fig. 1E).

### 3.2 TGF-β1-mediated fibrosis model

**Fig. 2:**
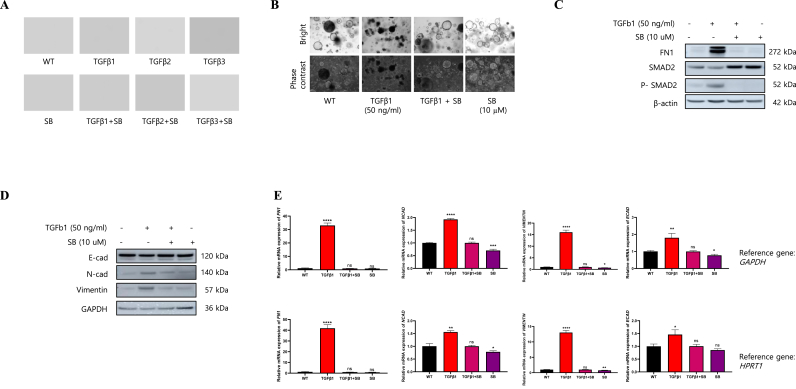
TGFβ1-induced fibrosis of alveolar organoids. (A) Bright-field images of alveolar type II lung organoids treated with TGF-β1, -2, and -3. (B) Bright-field images of alveolar organoids treated with TGF-β1 or SB431542. (C) Protein levels of FN1, SMAD2, *p*-SMAD2, and β-actin in the alveolar organoids. (D) Protein levels of E-cadherin, N-cadherin, vimentin, and GAPDH in the alveolar organoids. (E) Relative mRNA expression levels of *FN1*, *N-cadherin*, and *vimentin*, and *E-cadherin*.

To evaluate the response capacity of lung organoids to a fibrogenic stimulus,we treated lung organoid with TGF-β1, TGF-β2, and TGF-β3 for 48 h and analyzed the fibrotic changes. TGF-β1, TGF-β2, and TGF-β3 triggered rapid cell death and was blocked by TGF-βR1 inhibitor SB431542 (Fig. 2A and B). To better understand how TGF-β1induced fibrosis, we treated lung organoid with TGF-β1 for 24 h and analyzed the TGF-β1 signal pathway. We evaluated the levels of phosphorylated SMAD2 (*p*-SMAD2), a key downstream effector of TGF-β signaling. At the protein level, TGF-β1 increased the expression of fibronectinand phosphorylation of SMAD2 (Fig. 2C). In addition, TGF- β1 treatment enhanced the protein expression of mesenchymal markers such as N-cadherin and vimentin (Fig. 2D) and the mRNA expression levels of *N-cadherin* and *vimentin* were significantly higher than those in the control group (Fig. 2E). Whereas E-cadherin did not show any significant changes in the protein and mRNA expression levels (Fig. 2D and E). We found that TGF-β1 treatment induced fibrosis in lung organoids. Subsequent investigation revealed that cell demise occurred through apoptotic pathways (data not shown).

### 3.3 Identification of compounds that cause resistance to TGF-β1 in alveolar organoids

**Fig. 3:**
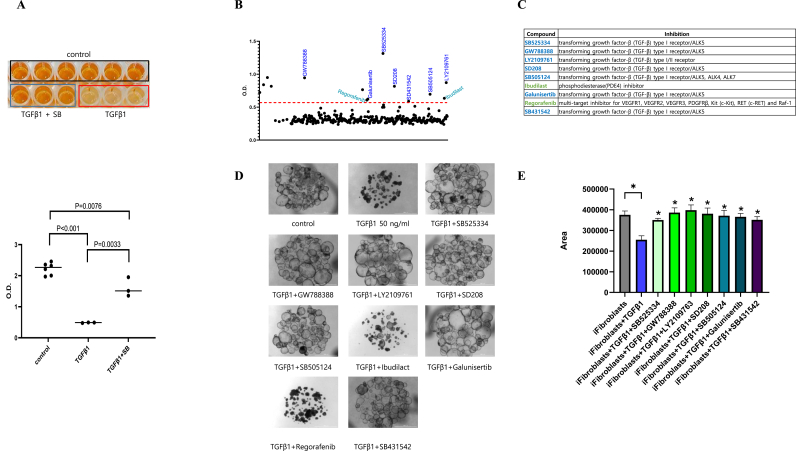
Validation of hits that conferred resistance to TGF-β1-induced viability in alveolar organoids (A) Picture and absorbance of WST-8 assay after 48 h exposure to TGF-β1 or SB431542. Absorbance was measured at 450 nm. n = 3 experiments. Student's *t*-test compared to control. (B) Absorbance of WST-8 assay after 48 h exposure to TGF-β1 or 320 anti-fibrosis compounds. Compounds were screen at a concentration of 10 μM. Hit drugs had higher optical density (OD) than negative drugs. The cut-off value (dashed line) is OD = 0.65 (C) The 10 hits among 320 antifibrosis compounds (D) Bright-field images of alveolar type II organoids treated with TGF-β1 or the 9 hit drugs. (E) Gel contraction assay was performed on fibroblasts using the 7 antifibrotic hit drug.

TGF-β1-induced viability of alveolar organoids was assayed in a 96-well plate using the Water-Soluble Tetrazolium 8 (WST-8) system (Fig. 3A).We identified 9 hit drugs that conferred resistance to TGF-β1-mediated viability in alveolar organoids. Most of the effective drugs were confirmed to be TGF-βR1 inhibitors (Fig. 3B and C). To validate the inhibitory effect on TGF-βR1, Matrigel embedded with alveolar organoids were treated with TGF-β1 in a 35 mm Petri dish. TGF-β1 triggered rapid cell death and was blocked by SB525334, GW788388, LY2109761, SD208, SB505124, Galunisertib, and SB431542, which were inhibitors of TGF-βR1. In contrast, ibudilast and regorafenib did not inhibit cell death (Fig. 3D).

### 3.4 Validation of hit compoundsusing gel contraction assay

A gel contraction assay was performed on fibroblasts using the 7 antifibrotic hit drugs. As expected, all the 7 drugs prevented gel contraction induced by TGF-β1, which indicates that these drugs can prevent TGF-βR1-induced fibrosis (Fig. 3E).

## 4 Discussion

TGF-β1 is a key mediator of fibrosis. Pirfenidone and nintedanib are commonly used for treating IPF and have the effect of suppressing lung fibrosis [9,21]. Although the exact mechanism of action is unknown, pirfenidone exhibits antifibrotic, anti-inflammatory, and antioxidant effects. It inhibits pulmonary fibrosis by suppressing TGF-β, which is involved in cell proliferation/differentiation, inhibiting collagen synthesis, and decreasing fibroblast proliferation [22]. Nintedanib, functioning as a tyrosine kinase inhibitor (TKI), simultaneously targets and inhibits receptors for several key growth factors involved in pulmonary fibrosis, including vascular endothelial growth factor (VEGF), fibroblast growth factor (FGF), and platelet-derived growth factor (PDGF) [23]. Both pirfenidone and nintedanib are effective in slowing the decline in lung function, reducing the risk of acute exacerbations, and increasing survival [23]. However, these treatments do not restore fibrotic lungs or prevent disease progression, and do not provide consistent benefits in terms of associated symptoms or quality of life. In our model, along with nintedanib and pirfenidone, we confirmed that the new pulmonary fibrosis-targeting drug could prevent TGF-β1-induced pulmonary fibrosis. In addition, we checked that they can treat pulmonary fibrosis after it has progressed to pulmonary fibrosis. Concomitant treatment with TGF-β1, nintedanib, and pirfenidone prevented lung fibrosis, similar to the effect of SB431542 treatment.We confirmed that treatment with TGF-β1 followed by treatment with SB431542 1 day later was effective in preventing lung fibrosis. This shows that TGF-βRI inhibitors such as SB431542 (Fig. 3D and E) are effective as therapeutic agents for treating pulmonary fibrosis. Galunisertib [24], which was evaluated in three clinical trials, is a TGF-βRI inhibitor, and could be a good treatment option for pulmonary fibrosis in the absence of any major side effects.

Our research highlights the considerable benefits of utilizing a 3D alveolar type II organoid model for drug screening, especially in the search for fibrosis inhibitors. By inducing fibrosis with TGF-β1, this model represents a significant advance over conventional 2D cell cultures, which simply cannot capture the intricate physiological environment of native tissues [24]. In conventional 2D drug screening systems, the reaction appears after a prolonged duration following the treatment of 2D cells with drugs, and validation further requires a considerable amount of time. However, in the 3D system, TGFβ1 causes morphological changes in the 3D alveolar organoids, which can be confirmed more easily than in the 2D system. We've made considerable progress in drug screening for fibrosis inhibitors with our 3D alveolar organoid model, which is induced by TGF-β1. Traditional 2D cell culture models (Table 2), despite their cost-effectiveness and suitability for high-throughput screening, fundamentally fall short in replicating the physiological complexity of native tissues [25,26]. In contrast, our organoid model faithfully reproduces the *in vivo* alveolar microenvironment, offering a more physiologically precise representation of how fibrosis progresses (Table 2). A unique advantage of our 3D model is the immediate and evident visual confirmation of fibrotic changes through standard bright-field microscopy, as illustrated in Fig. 2A and B. This provides a rapid, economical, and real-time phenotypic assessment, significantly enhancing the efficiency of initial drug candidate screening and increasing predictive accuracy. In addition, 3D culture systems display cell-to-matrix, cell-to-cell communication, and drug efficacy that is lacking in common 2D monolayer cultures [27]. Mimicking the lung microenvironment *in vitro* enables 3D systems to evaluate cell structure and function under pathologicaland homeostatic conditions. The antifibrotic drug library used in this study contained various drugs that prevent fibrosis. As the experiment used a TGF-β1 mediated fibrosis model, TGF-βRI inhibitors were accurately identified. In addition, while it takes a long time to confirm the antifibrotic effect in 2D, the 3D model can quickly confirm the effect within 2–3 days.

**Table 2:** Comparision of Reporter based TGF-β1 inhibitor screening and Organoid based TGF-β1 inhibitor screening.

Category	Reporter-Based TGF- β1 Screening	Organoid-Based TGF- β1 Screening
Screening Platform	Luciferase/Smad reporter assay	3D human stem cell derived organoids
Cell System Used	Single cell lines (e.g., HEK293, HaCaT, NIH-3T3)	Organ-mimicking multicellular clusters
Signal Detection Method	Smad3/4 activation → luciferase expression measurement	Immunofluorescence/RNA-seq based molecular response analysis
Throughput Level	High (96/384-well plate compatible)	Medium – improving for higher throughput
Physiological Relevance	Low – lacks complex cell-cell interactions	Very high – resembles actual tissue environments
3D Structural Representation	Not represented	Represented
Time/Cost Efficiency	Fast/Relatively low cost	High maintenance cost, slower growth
Drug Sensitivity	High – sensitive to TGF-β pathway inhibition	High – reflects tissue-level drug response
Quantification Capability	Very high – measurable luminescence signal	Moderate – requires additional processing

Furthermore, our 3D organoid model isn't just for drug discovery; it's also a powerful tool for investigating the fundamental mechanisms behind epithelial injury and repair in IPF. This is especially pertinent as the epithelial component's crucial role in IPF pathogenesis becomes increasingly recognized. Recent studies consistently underscore that epithelial-mesenchymal interactions are vital drivers of fibrosis progression, reinforcing the need for models that accurately reflect these dynamics. By faithfully reproducing these complex cellular interactions within a physiologically relevant context, our 3D alveolar organoid model provides an excellent platform to deepen our understanding of IPF progression and pinpoint new therapeutic targets [28]. By addressing this gap, our platform offers new insights into the pathophysiology of IPF and development of targeted therapeutic interventions (Fig. 2, Fig. 3D and E).

A core advantage of our alveolar organoid is its remarkable capacity to replicate the *in vivo* alveolar microenvironment with high accuracy. This faithfulness is critical for precisely mimicking how fibrosis progresses, particularly following epithelial injury. Given that alveolar type II cells are central to the lung's initial response to damage—and their malfunction is a key factor in fibrotic processes—our 3D model allows for direct observation of these complex cellular behaviors and the resulting fibrotic changes in a far more physiologically relevant setting than traditional flat cultures. Beyond its enhanced biological relevance, our model substantially improves the predictive accuracy of early-stage drug development for fibrosis. This is particularly true for therapies aimed at pathways triggered or worsened by epithelial damage.

Consistent with numerous studies demonstrating that TGF-β1 stimulation alters beta-actin expression [29], we also observed a similar phenomenon in our TGF-β1-induced alveolar organoid model in Fig. 1C. Beta-actin, a key component of the cytoskeleton, plays a crucial role in cell shape, motility, and tissue organization. This observed change in beta-actin expression in our organoids may be intrinsically linked to the dynamic morphological changes and cytoskeletal reorganization characteristic of TGF-β1-mediated fibrosis.

Despite our study's findings, future research should address several limitations. Firstly, this research depends on a single growth factor, TGF-β1, to induce lung fibrosis. It doesn't incorporate the complex interactions of various cell types (like fibroblasts, endothelial cells, and immune cells) or ECM components found *in vivo*. Consequently, the cellular responses we observed might not fully represent the complexity of a living organism. Secondly, there's a need for more in-depth studies on co-culture conditions that include a blood supply. In lung tissue, blood systems are vital for processes such as drug delivery, immune cell infiltration, and metastatic spread. Thirdly, comprehensive research on epigenetic regulation—including DNA methylation and histone modifications—within organoids is lacking. Understanding these mechanisms is crucial for comprehending long-term cell identity, gene expression patterns, and disease-related epigenetic changes.

In conclusion, investigating the response to lung epithelial injury using advanced models such as lung alveolar organoids enhances our understanding of pulmonary diseases and their treatment. Our study highlights the potential of alveolar organoids as a high-throughput screening platform for discovering antifibrotic drugs and studying the mechanisms of fibrosis regulation. This innovative approach may pave the way for more effective management of pulmonary diseases and the development of novel therapeutics targeting TGF-β1 signaling [30,31]. For our future study, we aim to comprehensively investigate the intricate interplay of diverse cell types, ECM components, circulatory systems, and epigenetic regulation (DNA methylation and histone modification) to uncover their combined effect on gene expression and cellular function.

## CRediT authorship contribution statement

**Hyeong-jun Han:** Writing – original draft, Visualization, Validation. **Hyunyoung Kim:** Funding acquisition, Project administration.

## Declaration of competing interest

All authors have read and approved the manuscript, and there are no potential conflicts of interest.

## Data Availability

sharing research data to evaluate my findings

## References

[1] Joo H., Min S., Cho S.W. (2024). Advanced lung organoids for respiratory system and pulmonary disease modeling. J. Tissue Eng..

[2] Liao D., Li H. (2020). Dissecting the niche for alveolar type II cells with alveolar organoids. Front. Cell Dev. Biol..

[3] Ng B. (2024). Interleukin-11 causes alveolar type 2 cell dysfunction and prevents alveolar regeneration. Nat. Commun..

[4] Kobayashi Y. (2020). Persistence of a regeneration-associated, transitional alveolar epithelial cell state in pulmonary fibrosis. Nat. Cell Biol..

[5] Jacob A. (2019). Derivation of self-renewing lung alveolar epithelial type II cells from human pluripotent stem cells. Nat. Protoc..

[6] Selman M., Pardo A. (2020). The leading role of epithelial cells in the pathogenesis of idiopathic pulmonary fibrosis. Cell. Signal..

[7] Moss B.J., Ryter S.W., Rosas I.O. (2022). Pathogenic mechanisms underlying idiopathic pulmonary fibrosis. Annu. Rev. Pathol..

[8] Kong J. (2021). Lung organoids, useful tools for investigating epithelial repair after lung injury. Stem Cell Res. Ther..

[9] Chianese M. (2024). Pirfenidone and nintedanib in pulmonary fibrosis: lights and shadows. Pharmaceuticals.

[10] Shi N. (2022). Research progress on drugs targeting the TGF-Beta signaling pathway in fibrotic diseases. Immunol. Res..

[11] Peng D. (2022). Targeting TGF-Beta signal transduction for fibrosis and cancer therapy. Mol. Cancer.

[12] Ali S. (2023). TGF-Beta signaling pathway: therapeutic targeting and potential for anti-cancer immunity. Eur. J. Pharmacol..

[13] Baba A.B. (2022). Transforming growth factor-beta (TGF-beta) signaling in Cancer-A betrayal within. Front. Pharmacol..

[14] Massague J., Sheppard D. (2023). TGF-Beta signaling in health and disease. Cell.

[15] Tie Y. (2022). TGF-Beta signal transduction: biology, function and therapy for diseases. Mol Biomed.

[16] Tzavlaki K., Moustakas A. (2020). TGF-Beta signaling. Biomolecules.

[17] Zhao B. (2024). A stepwise mode of TGFbeta-SMAD signaling and DNA methylation regulates naive-to-primed pluripotency and differentiation. Nat. Commun..

[18] Lee J.H., Massague J. (2022). TGF-Beta in developmental and fibrogenic EMTs. Semin. Cancer Biol..

[19] Park T.S. (2023). Protocol to generate endothelial cells, pericytes, and fibroblasts in one differentiation round from human-induced pluripotent stem cells. Star Protoc.

[20] Han H.J., Kim J.H. (2024). KSCBi005-A-10(hiPSC-HIF1alphaKO), a HIF1alpha knockout human induced pluripotent stem cell line, for demonstrating the role of cellular response to hypoxia. Stem Cell Res..

[21] Yang Y., Wang X., Zhang J. (2024). Pirfenidone and nintedanib attenuate pulmonary fibrosis in mice by inhibiting the expression of JAK2. J. Thorac. Dis..

[22] Ballester B., Milara J., Cortijo J. (2020). Pirfenidone anti-fibrotic effects are partially mediated by the inhibition of MUC1 bioactivation. Oncotarget.

[23] Yanalak A., Yazici O. (2025). Comparative long-term effects of nintedanib and pirfenidone in idiopathic pulmonary fibrosis: a real-life study with five-year Follow-up. Thorac Res Pract.

[24] Jing H. (2025). Recent advances in therapeutic use of transforming growth factor-beta inhibitors in cancer and fibrosis. Front. Oncol..

[25] Marvin D.L. (2022). Dynamic visualization of TGF-beta/SMAD3 transcriptional responses in single living cells. Cancers (Basel).

[26] Lohcharoenkal W. (2014). Luciferase reporter cells as a platform to detect SMAD-Dependent collagen production. J. Biosci. Bioeng..

[27] Vazquez-Armendariz A.I. (2022). 3D in vitro models: novel insights into idiopathic pulmonary fibrosis pathophysiology and drug screening. Cells.

[28] Niayesh-Mehr R. (2024). The role of epithelial-mesenchymal transition in pulmonary fibrosis: lessons from idiopathic pulmonary fibrosis and COVID-19. Cell Commun. Signal..

[29] Melchionna R. (2021). Actin cytoskeleton and regulation of TGFbeta signaling: exploring their links. Biomolecules.

[30] Chen S. (2024). ACTN1 promotes cell invasion, migration, and EMT in thyroid cancer and is associated with immune infiltration. Sci. Rep..

[31] Skora K. (2025). Analysis of the expression patterns of tumor necrosis factor alpha signaling pathways and regulatory MicroRNAs in astrocytic tumors. Int. J. Mol. Sci..

